# Weakly bound molecules as sensors of new gravitylike forces

**DOI:** 10.1038/s41598-019-51346-y

**Published:** 2019-10-15

**Authors:** Mateusz Borkowski, Alexei A. Buchachenko, Roman Ciuryło, Paul S. Julienne, Hirotaka Yamada, Yuu Kikuchi, Yosuke Takasu, Yoshiro Takahashi

**Affiliations:** 1 Institute of Physics, Faculty of Physics, Astronomy and Informatics, Nicolaus Copernicus University, Grudziadzka 5, 87-100 Torun, Poland; 2 Skolkovo Institute of Science and Technology, 100 Novaya Street, Skolkovo, Moscow Region 121205 Russia; 3 Institute of Problems of Chemical Physics RAS, Chernogolovka, Moscow Region 142432 Russia; 4 Joint Quantum Institute, NIST and the University of Maryland, College Park, Maryland 20742 USA; 5 Department of Physics, Graduate School of Science, Kyoto University, Kyoto, 606-8502 Japan

**Keywords:** Ultracold gases, Atomic and molecular interactions with photons, Laboratory astrophysics

## Abstract

Several extensions to the Standard Model of particle physics, including light dark matter candidates and unification theories predict deviations from Newton’s law of gravitation. For macroscopic distances, the inverse-square law of gravitation is well confirmed by astrophysical observations and laboratory experiments. At micrometer and shorter length scales, however, even the state-of-the-art constraints on deviations from gravitational interaction, whether provided by neutron scattering or precise measurements of forces between macroscopic bodies, are currently many orders of magnitude larger than gravity itself. Here we show that precision spectroscopy of weakly bound molecules can be used to constrain non-Newtonian interactions between atoms. A proof-of-principle demonstration using recent data from photoassociation spectroscopy of weakly bound Yb_2_ molecules yields constraints on these new interactions that are already close to state-of-the-art neutron scattering experiments. At the same time, with the development of the recently proposed optical molecular clocks, the neutron scattering constraints could be surpassed by at least two orders of magnitude.

## Introduction

The experimental search for non-Newtonian gravity has been taking place for years. Experimental bounds on hypothetical nanometer range forces could help verify several extensions to the Standard Model, including grand unification theories^1,2^, light dark matter models^3^ and extradimensional theories aimed at solving the hierarchy problem^4–6^. For two bodies of masses *m*_1_ and *m*_2_ separated by a distance *R*, a non-Newtonian correction to gravity is typically parameterised as an additional Yukawa-type potential^1,2,6^

1 $${V}_{NNG}=-\,\alpha G{m}_{1}{m}_{2}\frac{{e}^{-R/\lambda }}{R}\,.$$

In the particular case of new Yukawa-type “fifth forces” due to the exchange of light bosons that couple to nucleons^1–3,6,7^,

2 $${V}_{5}(R)=-{N}_{1}{N}_{2}\frac{{g}^{2}}{4\pi }\hslash c\frac{{e}^{-R/\lambda }}{R}\,,$$

where *N*_1,2_ are the atomic mass numbers, and the range *λ* = *ħ*/*Mc* is determined by the mass *M* of the new particle, while the dimensionless parameter *g*^2^ reflects the coupling strength between nucleons and the new particle field. In practice the constraints on *g*^2^ can be viewed as constraints on *α* and vice versa, with a conversion factor $\alpha \approx (\hslash c/4\pi G{m}_{p}^{2}){g}^{2}\approx 1.347\times {10}^{37}{g}^{2}$, where *m*_*p*_ is the proton mass^1^.

Experimental methods employed to provide bounds on *g*^2^ (or *α*) vary greatly depending on the range *λ* of the hypothetical new forces: from astrophysical observations^6^, to torsion balance experiments^2,8–11^, microcantilevers^12^, Casimir-less techniques^13,14^, atomic force microscopy^15^, and finally neutron scattering on a neutral atom target^7,16,17^. A promising technique based on direct comparison of spectroscopic measurements of deeply bound hydrogen molecules with precise *ab initio* calculations was also demonstrated^18^. While stringent for macroscopic ranges *λ*, the experimental constraints on corrections to gravity for tens of micrometers or less quickly become many orders of magnitude larger than gravity itself due to the presence of much stronger van der Waals or Casimir interactions at these length scales^14,19^.

**Figure 1:**
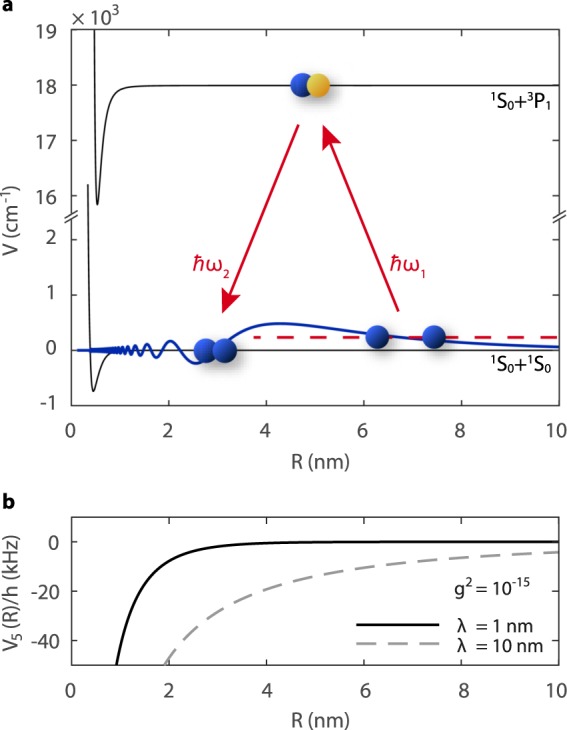
New gravitylike forces and long-range atomic interactions. (**a**) A schematic depiction of the principle of two color photoassociation spectroscopy. The vibrational wavefunction for a ^170^Yb_2_ bound state with a vibrational quantum number *v*′ = −2 (as counted from the dissociation limit) and total angular momentum *J* = 0 at a binding energy ${E}_{b}/h=-\,463.72552(80)$ MHz peaks at *R* ≈ 4.2 nm (blue). (**b**) Example Yukawa-type gravitylike potentials *V*_5_(*R*) for two ^170^Yb atoms, as defined by Eq. (2).

Here we propose to search for new gravitylike forces in long range atomic interactions using high precision spectroscopy of weakly bound ultracold molecules. Unlike deeply bound hydrogen dimers^18^, where the equilibrium distance lies at *R* ≈ 0.074 nm, the vibrational motion in bound states close to a molecule’s dissociation limit can extend to several nanometers (Fig. 1a)^20^. The presence of a new Yukawa-type potential could be manifested as a perturbation to near-threshold vibrational series. For *g*^2^ = 10^−15^, comparable to current limits in the nanometer range^7^, *V*_5_(*R*) could contribute an additional several to several tens of kHz to the interaction potential for *R* comparable to the size of the molecule (Fig. 1b). Weakly bound molecules composed of bosonic two-valence-electron atoms, like Yb, Sr, or Hg, have simple rovibrational structure thanks to their spin-singlet electronic ground state and a lack of hyperfine structure. Near-threshold vibrational splittings depend chiefly on the dominant long range *R*^−6^ van der Waals interaction and are to a large extent insensitive to the details of the short range potential^20,21^. The narrow intercombination lines present in divalent species facilitate measurements of the positions of near-threshold bound states of Yb_2_^22^ and Sr_2_^23,24^ to an already impressive sub-kHz accuracy which in the future could further be improved by several orders of magnitude using lattice clock techniques^25^. Thus, weakly bound molecules composed of Yb or Sr atoms make excellent testing grounds in the search for new interactions by uniting precision measurements with a relatively simple level structure.

## Weakly Bound Molecules as a Testbed for Non-Newtonian Gravity

We demonstrate our proposal by carrying out a proof-of-concept determination of constraints on the new forces using the recent state-of-the-art measurements of near-threshold Yb_2_ bound state energies^22^. With a total of 13 measured rovibrational state positions (Table 1) for three Yb_2_ isotopomers it is the largest of the currently available sub-kHz datasets. The bound state energies were measured using two-color photoassociation spectroscopy^20^ of Yb Bose-Einstein condensates in an optical dipole trap. Here, two lasers were used to induce Raman coupling between colliding atomic pairs and a rovibrational level in the electronic ground state using an intermediate excited state. Once the difference in the laser frequencies $\hslash {\omega }_{1}-\hslash {\omega }_{2}$ matched the energy *E*_*b*_ of a vibrational level in the electronic ground state with respect to the dissociation limit, loss of atoms from the trap was observed. Systematic shifts from the trapping and photoassociation lasers and the mean-field shift of the BECs have been taken into account leading to experimental uncertainties ≈500 Hz for most bound state energies.

**Table 1:** Vibrational state positions for ground state Yb_2_ molecules^22^.

Isotope	*v*′	*J*	*E*_*b*_ (MHz)	*δE*_*b*_(*λ*, *g*^2^) (kHz)
^168^Yb	−2	2	−145.53196(48)	−0.02
^168^Yb	−2	0	−195.18141(46)	−1.59
^170^Yb	−1	2	−3.66831(32)	−16.78
^170^Yb	−1	0	−27.70024(44)	−17.60
^170^Yb	−2	2	−398.05626(46)	+12.53
^170^Yb	−2	0	−463.72552(80)	+8.96
^170^Yb	−3	2	−1817.14074(80)	−1.79
^170^Yb	−2	0	−1922.01467(505)	−10.39
^174^Yb	−1	0	−10.62513(53)	−19.60
^174^Yb	−2	2	−268.63656(56)	+3.64
^174^Yb	−2	0	−325.66378(98)	+0.98
^174^Yb	−3	2	−1432.82653(75)	+4.77
^174^Yb	−3	0	−1527.88543(34)	−2.70

All bound state positions are given in MHz with respect to the ^1^S_0_ + ^1^S_0_ dissociation limit. The quantum numbers *v*′ and *J* correspond to, respectively, the vibrational quantum number (counted from the dissociation limit), and the total angular momentum. Values in parentheses are standard uncertainties. We also show an example shift $\delta {E}_{b}(\lambda ,\alpha )$ to the fitted theoretical energies due to extra Yukawa-type interactions [Eq. (2)] for $\lambda =1\,{\rm{nm}}$ and ${g}^{2}=1.9\times {10}^{-15}$, our extracted 95% CI limit after other parameters of the fit are optimized.

**Figure 2:**
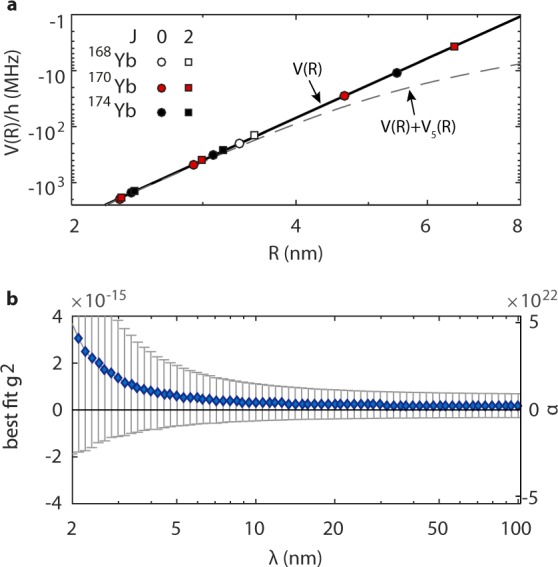
Sensitivity of long range atomic interactions to new gravitylike forces. (**a**) Long range *R*^−6^ van der Waals interaction between two Yb atoms (in log scale). Markers indicate positions of bound states measured in photoassociative spectroscopy^22^. Dashed line shows the same potential modified by an additional Yukawa interaction for ${g}^{2}={10}^{-13}$ (much larger than current limits at nanometers to make it visible in plot) and $\lambda =1$ nm. (**b**) Best fit coupling parameters *g*^2^ as a function of *λ*. All of the fitted *g*^2^ values are compatible with zero (horizontal line) well within one standard uncertainty (shown as error bars).

The measured binding energy range of −1922 to −3.7 MHz corresponds to classical outer turning points in between *R* = 2.3 and *R* = 6.5 nm (Fig. 2a). At these internuclear distances the atomic potential is dominated by the long range *R*^−6^ van der Waals interaction. Adding the Yukawa-type potential *V*_5_(*R*) imposes a significant change to the long range atomic interaction that can be reliably distinguished from the expected *R*^−6^ behavior. For example, in Table I we show shifts *δE*_*b*_ to theoretical bound state energies for *λ* = 10 nm and *g*^2^ = 1.9 × 10^−15^ after other parameters of our interaction model (see below) are optimized to the experimental data. The additional Yukawa potential tends to systematically lower the energies of most weakly bound states in a manner that cannot be compensated for by changing the long range interaction parameters.

We describe the interactions between two Yb atoms using our previous mass-scaled interaction model^22^. For a total angular momentum *J* the rovibrational level energies obey the radial Schrödinger equation,

3 $$(-\frac{{\hslash }^{2}}{2\mu }\frac{{d}^{2}}{d{R}^{2}}+V(R)+{V}_{{\rm{ad}}}(R)+\frac{{\hslash }^{2}J(J+1)}{2\mu {R}^{2}})\Psi (R)={E}_{b}\Psi (R)\,.$$

Since both atoms are in structureless ^1^S_0_ electronic ground states, there are no permanent multipole moments and at large separations the atoms interact purely due to dispersion. The long range part of the interaction potential *V*(*R*) is dominated by the induced dipole-dipole ${C}_{6}{R}^{-6}$ term, with ${C}_{6}=1937.27(57)\,{E}_{h}{a}_{0}^{6}$ ^22^ (the Hartree energy ${E}_{h}\approx 4.359744650(54)\times {10}^{-18}$ J and Bohr radius ${a}_{0}\approx 5.2917721067(12)\times {10}^{-11}$ m are the atomic units of energy and distance). The next dispersion term *C*_8_*R*^−8^, with ${C}_{8}=2.265(17)\times {10}^{5}\,{E}_{h}{a}_{0}^{8}$, describes the induced dipole-quadrupole interaction. Although at *R* = 5 nm it represents just 1.3% of the potential energy, it is critical to reach proper quality of the fit. On the other hand, introducing the next dispersion term, *C*_10_*R*^−10^, no longer improves the fit showing that the measured bound states are insensitive to it due to their extended classical outer turning points. No prior *ab initio* prediction for the value of *C*_10_ exists, so we leave it out of the model. Similarly, no improvement is seen when introducing the Casimir-Polder effect, whether by directly implementing *ab initio* corrections to the long range potential^26^ or adding a fitted +*w*_4_*R*^−4^ term. The analytic dispersive interaction $V(R)\to -{C}_{6}{R}^{-6}-{C}_{8}{R}^{-8}$ is smoothly connected to a realistic *ab initio* short range potential using a smooth transition function^27^. The best fit potential depth ${D}_{e}=739.73(60)\,{{\rm{cm}}}^{-1}$ is set by scaling the *ab initio* potential by just 2.3%. Our interaction model also includes two beyond-Born-Oppenheimer effects – the adiabatic correction ${V}_{{\rm{ad}}}(R)$ as calculated by Lutz and Hutson^28^ and an *R*-dependent effective reduced mass *μ*^29^. The latter is a nonadiabatic effect and is modeled by having the reduced mass *μ* vary smoothly between half the nuclear mass for *R* → 0 and half the atomic mass when the two atoms are well separated (*R* → ∞)^22^. The parameters *C*_6_, *C*_8_ and *D*_*e*_ are fitted to the experimental data by nonlinear least squares. The van der Waals parameters *C*_6_, and to a lesser extent *C*_8_, determine the near-threshold vibrational spacings, whereas the depth *D*_*e*_ fixes the phase of the short range radial wavefunction and, by proxy, the position of the entire near-threshold vibrational spectrum^20,21^. Despite the simple, purely electrostatic model of the long range interaction that lacks quantum electrodynamic (QED) and relativistic corrections, our model still reproduces the positions of near-threshold bound states in the Yb_2_ molecule to ≈30 kHz.

## Determination of Constraints

**Figure 3:**
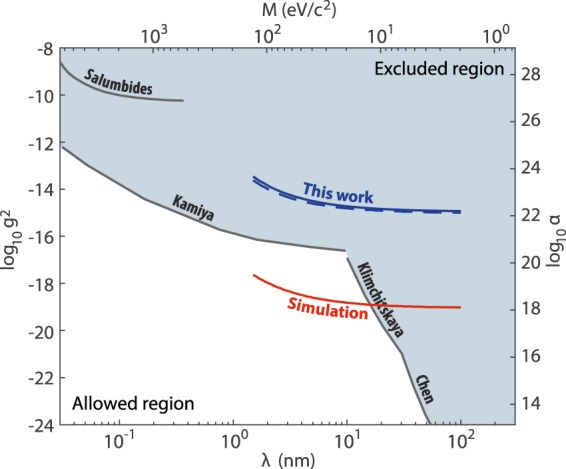
Constraints on Yukawa-type gravitylike forces. Feldman-Cousins limits on *g*^2^ as a function of Yukawa range $\lambda =\hslash /Mc$, where *M* is the mass of the hypothetical new particle, and comparison with other constraints derived from spectroscopy of hydrogen molecules (Salumbides *et al*.^18^), neutron scattering (Kamiya *et al*.^7^), atomic force microscopy (Klimchitskaya *et al*.^15^) and a Casimir-less experiment (Chen *et al*.^14^).

We first extract our limits on the magnitude of the coupling parameter *g*^2^ using the complete dataset. We add the new interaction ${V}_{5}(R)$ to the Hamiltonian in Eq. (3) and run a series of least-squares fits for varying Yukawa ranges *λ*. In each fit the *λ* parameter is held fixed, whereas the three adjustable potential parameters, *C*_6_, *C*_8_, and *D*_*e*_^22^ and now also the coupling *g*^2^, are optimized again using nonlinear least-squares. Even though an independent prediction based on atomic polarizabilities for ${C}_{6}=1929(39)$^30^ exists, its error bar is much larger than the statistical uncertainty of our theoretical fits, so we can not use it to constrain our fits appreciably. We also find that the re-fitted *C*_6_ values differ from the original fit by at most 0.5%, depending on *λ*. The uncertainties for the four fitted parameters are scaled by the factor $\sqrt{{\chi }^{2}/{\rm{d}}{\rm{o}}{\rm{f}}}$, where ${\rm{d}}{\rm{o}}{\rm{f}}=13-4-1=8$ is the number of degrees of freedom to take into account the possible systematic error of the theoretical model. For our dataset the fits converge reliably for *λ* in the range of $2\ldots 100$ nm. The resulting *g*^2^ values are all compatible with zero well within one standard uncertainty (Fig. 2b). The other fit parameters remain in agreement with their original values. For example, for a Yukawa range of $\lambda =10\,{\rm{nm}}$, ${C}_{6}=1937.15(46)\,{E}_{h}{a}_{0}^{6}$, ${C}_{8}=2.264(13)\times {10}^{5}\,{E}_{h}{a}_{0}^{8}$, and ${D}_{e}=739.76(43)\,{{\rm{cm}}}^{-1}$, whereas ${\chi }^{2}=9532$, slightly below the ${\chi }^{2}=9555$ of the original fit^22^. Following Kamiya *et al*.^7^, we determine the 95% confidence limits (Fig. 3) using the Feldman-Cousins approach^31^ which takes into account the fact that *g*^2^ should have a non-negative value. Secondly, we verify that our constraints are due to the impact the Yukawa potential has on long range interactions, rather than its dependence on the number of nucleons. To do so, we have repeated our fitting procedure but with the dataset restricted to ^170^Yb_2_. Only for this isotope a sufficient number of experimental data points is available to allow a convincing fit for four fitted parameters (${\rm{dof}}=6-4-1=1$). Finally, we run a projection for a hypothetical scenario where theory could fit experimental data to within 1 Hz. The state-of-the-art measurements of bound state positions in weakly bound molecules reach an accuracy of hundreds of Hz^22–24^, which may in the near future be improved by several orders of magnitude using molecular clock transitions^25^. Atomic optical clocks currently have short-term relative instabilities of about 10^−15^ (∼1 Hz absolute), and with proper averaging reach a relative accuracy of 10^−18^. Thus, sub-Hz-level measurements of molecular level positions could reasonably be attainable. Conversely, with improved description of the long range interactions the constraints on new gravitylike forces could improve by several orders of magnitude. To obtain the projected constraint (“Simulation” in Fig. 3) we used a simulated dataset, comprised of theoretical bound state positions for the same bound states as listed in Table 1, calculated using our original theoretical interaction model^22^ to which we added a Gaussian noise with a standard deviation of 1 Hz to simulate experimental uncertainties. Thus, this is a test of the sensitivity of our method for a case where the theory is sufficiently complete, and any discrepancies between the theoretical fit and experimental data is dominated by experimental uncertainties.

The constraints obtained for the current photoassociative dataset are already close to the current state-of-the-art. For a Yukawa range of $\lambda =10\,{\rm{nm}}$ the best fit coupling is ${g}^{2}=(3.2\pm 7.9)\times {10}^{-16}$, which corresponds to a Feldman-Cousins 95% confidence level limit of ${g}^{2}\le 1.9\times {10}^{-15}$, just two orders of magnitude above the neutron scattering constraints of Kamiya *et al*.^7^. Restricting the dataset to ^170^Yb_2_ results in nearly identical, and even slightly more stringent constraints. This can be explained by the fact that it is easier to accurately reproduce the photoassociation spectra for a single isotope than to construct a fully mass-scaled model^22^. When limited to one isotope, the model can fit the photoassociation data to within about 10 kHz, rather than the 30 kHz for a mass-scaled model. This shows that it may be a better strategy to measure many lines for a single isotope (e.g. for many rotational levels) rather than use many isotopes. At a certain level of accuracy it will be necessary to take into account e.g. the slight isotopic dependence of the van der Waals coefficients^32,33^ or the potential depth and may require separate fitting parameters for each isotope. This problem could be mitigated by calculating the small isotopic differences using *ab initio* methods while leaving the isotope-independent value as a fitted parameter. The few-percent relative accuracy typical for *ab initio* calculations for heavy dimers may suffice for the small corrections.

Our projected constraints for a hypothetical scenario, where theory matches experiment to within 1 Hz, show a significant potential for our method. For instance, the current limits for $\lambda =2$ nm to $\lambda =10$ nm could be surpassed by about 1.5–2 orders of magnitude. This, however, will also require the inclusion of several subtle QED and relativistic effects^26,32,34^ in the theoretical description of long range atomic interactions. If data for many isotopes are to be used^35,36^ an *ab initio* calculation of isotope-dependent corrections, like the adiabatic, nonadiabatic or nuclear volume corrections^28^ may prove necessary. Although our method only tests the presence of a Yukawa-type potential, in the future, the mass-dependency of Yukawa gravitylike forces could additionally constrain their magnitude, through its impact on the mass-scaling behavior of the near-threshold bound state positions between different isotopes. Even shorter range forces, where *λ* is much smaller than the ranges investigated here, could impact the phase of the short range wavefunction in a detectable manner^28^. Such attempts, however, will require a full understanding of the mass-dependent Beyond-Born-Oppenheimer corrections^22,28,29,33^.

## Conclusion and Outlook

In conclusion, we have proposed and demonstrated a new method for constraining new Yukawa-type gravitylike forces in the nanometer range based on precision spectroscopy of near-threshold molecular states. Ultracold weakly bound molecules composed of ground state spin-singlet atoms, like Yb or Sr, are an excellent testing ground in searching for new interactions thanks to their simple structure and narrow optical transitions that allow for precision measurements. The available photoassociation data^22^ for the Yb_2_ molecule already makes it possible to derive constraints on new nanometer range Yukawa-type forces close to current state-of-the-art constraints derived from mature experimental techniques like neutron scattering^7^ or measurements of Casimir-Polder forces^15^. Our method is complementary to the spectroscopy of deeply bound hydrogen molecules (Salumbides *et al*.^18^), as it excels for Yukawa ranges of several nanometers, complementing the range of ∼0.1 nm probed in the latter. In the future, with the development of next-generation optical molecular clocks^25,35^ and with improved theoretical description of long range interactions^32,34^, our technique could constrain new gravitylike forces at unprecedented levels and provide a valuable means of testing new physics beyond the Standard Model^1–6^.

## Data Availability

The data supporting the findings of this study are available within the paper and references therein.

## References

[1] Fayet P (1996). New interactions and the standard models. Classical and Quantum Gravity.

[2] Adelberger EG, Gundlach JH, Heckel BR, Hoedl S, Schlamminger S (2009). Torsion balance experiments: A low-energy frontier of particle physics. Progress in Particle and Nuclear Physics.

[3] Knapen S, Lin T, Zurek KM (2017). Light dark matter: Models and constraints. Physical Review D.

[4] Arkani-Hamed N, Dimopoulos S, Dvali G (1998). The hierarchy problem and new dimensions at a millimeter. Physics Letters B.

[5] Antoniadis I, Arkani-Hamed N, Dimopoulos S, Dvali G (1998). New dimensions at a millimeter to a fermi and superstrings at a TeV. Physics Letters B.

[6] Adelberger EG, Heckel BR, Nelson AE (2003). Tests of the Gravitational Inverse-Square Law. Annual Review of Nuclear and Particle Science.

[7] Kamiya Y, Itagaki K, Tani M, Kim GN, Komamiya S (2015). Constraints on new gravitylike forces in the nanometer range. Physical Review Letters.

[8] Su Y (1994). New tests of the universality of free fall. Phys. Rev. D.

[9] Bordag M, Mohideen U, Mostepanenko VM (2001). New developments in the Casimir effect. Physics Reports.

[10] Masuda M, Sasaki M (2009). Limits on Nonstandard Forces in the Submicrometer Range. Physical Review Letters.

[11] Sushkov AO, Kim WJ, Dalvit DAR, Lamoreaux SK (2011). New Experimental Limits on Non-Newtonian Forces in the Micrometer Range. Physical Review Letters.

[12] Geraci AA, Smullin SJ, Weld DM, Chiaverini J, Kapitulnik A (2008). Improved constraints on non-Newtonian forces at 10 microns. Physical Review D.

[13] Decca RS (2005). Constraining New Forces in the Casimir Regime Using the Isoelectronic Technique. Physical Review Letters.

[14] Chen Y-J (2016). Stronger Limits on Hypothetical Yukawa Interactions in the 30–8000 nm Range. Physical Review Letters.

[15] Klimchitskaya GL, Mohideen U, Mostepanenko VM (2013). Constraints on corrections to Newtonian gravity from two recent measurements of the Casimir interaction between metallic surfaces. Physical Review D.

[16] Pokotilovski YN (2006). Constraints on new interactions from neutron scattering experiments. Physics of Atomic Nuclei.

[17] Nesvizhevsky VV, Pignol G, Protasov KV (2008). Neutron scattering and extra-short-range interactions. Physical Review D.

[18] Salumbides EJ (2013). Bounds on fifth forces from precision measurements on molecules. Physical Review D.

[19] Kapner DJ (2007). Tests of the Gravitational Inverse-Square Law below the Dark-Energy Length Scale. Physical Review Letters.

[20] Jones KM, Tiesinga E, Lett PD, Julienne PS (2006). Ultracold photoassociation spectroscopy: Long-range molecules and atomic scattering. Reviews of Modern Physics.

[21] Le Roy RJ, Bernstein RB (1970). Dissociation Energy and Long-Range Potential of Diatomic Molecules from Vibrational Spacings of Higher Levels. The Journal of Chemical Physics.

[22] Borkowski M (2017). Beyond-Born-Oppenheimer effects in sub-kHz-precision photoassociation spectroscopy of ytterbium atoms. Phys. Rev. A.

[23] Stellmer S, Pasquiou B, Grimm R, Schreck F (2012). Creation of Ultracold Sr2 Molecules in the Electronic Ground State. Physical Review Letters.

[24] McGuyer BH (2015). High-precision spectroscopy of ultracold molecules in an optical lattice. New Journal of Physics.

[25] Borkowski M (2018). Optical Lattice Clocks with Weakly Bound Molecules. Physical Review Letters.

[26] Zhang P, Dalgarno A (2008). Long-range interactions of ytterbium atoms. Molecular Physics.

[27] Janssen LMC, Groenenboom GC, Avoird AVD, Żuchowski PS, Podeszwa R (2009). Ab initio potential energy surfaces for with analytical long range. The Journal of Chemical Physics.

[28] Lutz JJ, Hutson JM (2016). Deviations from Born-Oppenheimer mass scaling in spectroscopy and ultracold molecular physics. Journal of Molecular Spectroscopy.

[29] Pachucki K, Komasa J (2008). Nonadiabatic corrections to the wave function and energy. The Journal of Chemical Physics.

[30] Safronova, M. S., Porsev, S. G. & Clark, C. W. Ytterbium in quantum gases and atomic clocks: Van der waals interactions and blackbody shifts. *Physical Review Letters* **109**, 230802, arXiv:1208.1456v1 (2012). 10.1103/PhysRevLett.109.230802 23368178

[31] Feldman GJ, Cousins RD (1998). Unified approach to the classical statistical analysis of small signals. Physical Review D.

[32] Moszyński R, Łach G, Jaszuński M, Bussery-Honvault B (2003). Long-range relativistic interactions in the Cowan-Griffin approximation and their QED retardation: Application to helium, calcium, and cadmium dimers. Physical Review A.

[33] Przybytek M, Jeziorski B (2012). Long-range asymptotic expansion of the diagonal Born-Oppenheimer correction. Chemical Physics.

[34] Balcerzak JG, Lesiuk M, Moszynski R (2017). Calculation of Araki-Sucher correction for manyelectron systems. Physical Review A.

[35] Borkowski M, Buchachenko A A, Ciuryło R, Julienne P S, Yamada H, Yuu K, Takahashi K, Takasu Y, Takahashi Y (2017). Probing Non-Newtonian gravity by photoassociation spectroscopy. Journal of Physics: Conference Series.

[36] Kondov SS (2019). Molecular lattice clock with long vibrational coherence. Nature Physics.

