# Author Correction: Weakly bound molecules as sensors of new gravitylike forces

**DOI:** 10.1038/s41598-020-58617-z

**Published:** 2020-01-28

**Authors:** Mateusz Borkowski, Alexei A. Buchachenko, Roman Ciuryło, Paul S. Julienne, Hirotaka Yamada, Yuu Kikuchi, Yosuke Takasu, Yoshiro Takahashi

**Affiliations:** 1 Institute of Physics, Faculty of Physics, Astronomy and Informatics, Nicolaus Copernicus University, Grudziadzka 5, 87-100 Torun, Poland; 2 Skolkovo Institute of Science and Technology, 100 Novaya Street, Skolkovo, Moscow Region 121205 Russia; 3 Institute of Problems of Chemical Physics RAS, Chernogolovka, Moscow Region 142432 Russia; 4 Joint Quantum Institute, NIST and the University of Maryland, College Park, Maryland 20742 USA; 5 Department of Physics, Graduate School of Science, Kyoto University, Kyoto, 606-8502 Japan

Correction to: *Scientific Reports* 10.1038/s41598-019-51346-y, published online 15 October 2019

This Article contains errors in the text under subheading ‘Determination of Constraints’.

“If data for many isotopes are to be used^35,36^ an *ab initio* calculation of isotope-dependent corrections, like the adiabatic, nonadiabatic or nuclear volume corrections^28^ may prove necessary.”

should read:

“If data for many isotopes are to be used^35^ an *ab initio* calculation of isotope-dependent corrections, like the adiabatic, nonadiabatic or nuclear volume corrections^28^ may prove necessary.”

In addition,

“In the future, with the development of next-generation optical molecular clocks^25,35^ and with improved theoretical description of long range interactions^32,34^, our technique could constrain new gravitylike forces at unprecedented levels and provide a valuable means of testing new physics beyond the Standard Model^1,2,3,4,5,6^.”

should read:

“In the future, with the development of next-generation optical molecular clocks^25,36^ and with improved theoretical description of long range interactions^32,34^, our technique could constrain new gravitylike forces at unprecedented levels and provide a valuable means of testing new physics beyond the Standard Model^1,2,3,4,5,6^.”

This Article also contains an error in Reference 27 which was incorrectly given as:

“Janssen, L. M. C., Groenenboom, G. C., Avoird, A. V. D., Żuchowski, P. S. & Podeszwa, R. *Ab initio* potential energy surfaces for with analytical long range. *The Journal of Chemical Physics* **131**, 224314 (2009).”

The correct reference is listed below:

“Janssen, L. M. C., Groenenboom, G. C., Avoird, A. V. D., Żuchowski, P. S. & Podeszwa, R. *Ab initio* potential energy surfaces for NH(^3^∑^−^)-NH(^3^∑^−^) with analytical long range. *The Journal of Chemical Physics* **131**, 224314 (2009).”

